# A Metagenomic Survey of Virological Hazards in Market-Ready Oysters

**DOI:** 10.1007/s12560-024-09630-2

**Published:** 2025-01-10

**Authors:** René A. M. Dirks, Nils P. Sosef, Johanna T. M. Zwartkruis-Nahuis, Marijke M. A. Thijssen, Claudia C. C. Jansen, Ingeborg L. A. Boxman

**Affiliations:** Wageningen Food Safety Research, Akkermaalsbos 2, 6708 WB Wageningen, the Netherlands

**Keywords:** Food safety, Viral contamination, Virome, Metagenome, Shellfish, Oyster

## Abstract

Viral contamination of bivalve molluscs, such as oysters, is a well-recognized food safety risk. The aim of this study was to assess virological hazards in market-ready oysters on the Dutch market. Non-targeted metagenome analysis was first performed on norovirus spiked-in samples showing linear and sensitive detection of norovirus GI.2 and GII.4 down to 14 and 5 genome copies per reaction, respectively. Subsequently, metagenomic measurements were performed to detect vertebrate viral genomes present in 24 undepurated B-area samples and 144 market-ready oyster samples taken in November up to and including February of the years 2015–2021. Genome sequences from fifteen viral species were identified in market-ready oysters which are associated with infections in humans and were detected above the genomic coverage threshold (5%) applied. Among these, the two genera from the *Caliciviridae* family, norovirus and sapovirus were detected at high prevalence (44 and 30%). Additionally, adeno-associated dependoparvovirus A and B as well as Aichi virus A and B (ribo)nucleic acids were detected (42, 33, 6, and 11%). Nucleic acids from virus species in oysters included potentially hazardous *Picobirnavirus, Anellovirus,* and multiple *Circoviridae* and *Genomoviridae* species. By integrating metagenome analysis into the monitoring process, researchers, food producers and regulatory bodies can gain valuable insights into the viral communities present in the food chain. This allows for the detection of potential pathogenic hazards at an early stage, providing an opportunity for tailored monitoring programs and targeted interventions to maintain the sanitary quality of the production area and safeguard public health.

**Supplementary Information:**

The online version contains supplementary material available at 10.1007/s12560-024-09630-2.

## Introduction

Bivalve molluscs, particularly oysters and mussels, represent a significant food source. These organisms play a crucial role in marine ecosystems through their filter-feeding behavior (Jiang et al., 2023). This, however, also results in bioaccumulation of e.g., bacteria, viruses, biotoxins, or heavy metals as recently reviewed (Barchiesi et al., 2020; Fehrenbach et al., 2024). The presence of micro-organisms pose a well-recognized food safety risk especially when these shellfish are consumed raw or undercooked (Savini et al., 2021). To date, regulations for the microbiological safety of bivalve molluscs are based on bacterial indicators for fecal contamination in shellfish meat (e.g., Europe) (Commission-Regulation (EC), 2008), or growing waters (e.g., United States) (FDA, 2015). Classification of the growing areas based on results of sanitary surveys and bacteriological testing, determines whether harvested bivalve molluscs can be sent for direct human consumption or require post-harvest treatment such as purification, relaying or post-harvest cooking (Gyawali et al., 2019).

Despite these regulations, numerous oyster related illnesses have been reported, predominantly caused by human viruses, such as, human norovirus causing gastroenteritis and hepatitis A virus leading to acute hepatitis (Bellou et al., 2013). Correlations between levels of norovirus with either *Escherichia coli* (*E. coli*) or total coliforms have been shown to be poor (Dirks et al., 2021; Younger et al., 2018), whereas recent studies have highlighted the limitations of depuration concerning viral contaminations. For instance, investigations into the effectiveness of depuration for human norovirus (Battistini et al., 2021; Martinez-Albores et al., 2020; McLeod et al., 2017; Rupnik et al., 2021) and hepatitis A virus (Heller et al., 1986; Love et al., 2010; Polo et al., 2014) have demonstrated that these viruses exhibit a higher persistence compared to *E. coli*, even after one or two days of depuration. In addition, viral levels after treatment will also be dependent on initial viral loads (Rupnik et al., 2021).

To date, no microbiological criteria have been set for human noroviruses in bivalve molluscs. The prevalence of human norovirus RNA in market-ready oysters harvested from European waters is high, even exceeding 30% in some studies (Dirks et al., 2021; EFSA, 2019). Besides norovirus and hepatitis A virus, other viral pathogens, such as, Aichi virus, adenovirus, astrovirus, enterovirus, hepatitis E virus and rotavirus, have also been detected in bivalve molluscs in monitoring studies (Boxman, 2010; Desdouits et al., 2023), as well as in outbreaks investigations (Le Guyader et al., 2008).

A comprehensive assessment of the viral hazards present in market-ready oysters from North-Western Europe has not yet been conducted. An exhaustive hazard assessment could provide insight to food producers and risk managers involved in food safety and will thereby benefit public health. Metagenomics, also called viromics when pertaining specifically to viruses, offers a promising approach for the simultaneous detection of a wide array of viral pathogens without prior knowledge of the specific viruses present (Ramamurthy et al., 2017). This molecular fingerprinting technique enables the identification of potential virological hazards from food in a single analysis (Desdouits et al., 2020). However, the presence of interfering substances, such as matrix genomic material and non-relevant microbiota, can complicate metagenomic measurements, rendering them less effective and more costly (Billington et al., 2022). To address this issue, hybridization capture techniques have been developed (Briese et al., 2015). These techniques utilize hybridization panels designed to target a broad spectrum of known viral molecules, enhancing the specificity and efficiency of the metagenomic analysis. Previous studies employing metagenomics for bivalve molluscan analysis have demonstrated the potential of this technique (Bonny et al., 2021; Ollivier et al., 2022; Strubbia et al., 2019; Tan et al., 2021).

Building on these foundational studies, we set out to provide a comprehensive and systematic characterization of viral RNA and DNA in market-ready oysters on the Dutch market using the hybridization capture method. First, we determined the sensitivity and linearity parameters for human norovirus GI and norovirus GII, followed by assessment of the viral nucleic acid capture efficiency when applied to mixtures of viruses. The capability to detect a diversity of viruses present in the environment was demonstrated using a set of 24 bivalve molluscan samples requiring post-harvest treatment. Finally, a metagenomic analysis was conducted on 144 retail oysters collected in the Netherlands in the period November 2015-February 2021, in months with high gastroenteric virus activity (November up to and including February). The results were further verified by subtyping of known gastroenteric agents, and (RT-)digital PCR assays for a panel of the viruses detected.

## Material and Methods

### Sampling and Selection

Sampling of oysters (species *Magallana gigas*) for viral analyses was performed by Inspectors of the Netherlands Food and Consumer Product Safety Authority (NVWA) as part of a monitoring program. Sampling numbers and locations varied upon prioritization by the authority. Market-ready oysters were collected at Dutch dispatch centers from sorting and packaging lines as well as packed products collected at retail stores in the Netherlands. Each oyster sample consisted of at least 10 animals. Samples were kept refrigerated (4 °C) during transport for direct delivery at the laboratory of Wageningen Food Safety Research (WFSR).

All samples were dissected and extracted according to ISO 15216–1:2017, as described previously (Dirks et al., 2021). In brief, for each sample, 10 or more individual animals were shucked and the gastrointestinal tissue was isolated by dissection and finely chopped with a razor blade. Two grams of the chopped tissues were incubated with feline calicivirus (2 × 10^3^ TCID_50_) as process control virus and viruses were dissociated from the matrix using proteinase K treatment and clarification. After viral analysis, the remainder of proteinase K-treated digestive tissues were archived and stored in − 80 °C.

For this study, 261 archived proteinase K-treated digestive tissues were available from oysters that had been collected in the period from November 2015 up to and including February 2021, varying from 24 to 69 samples for each period from November up to and including February. To obtain a representative set of samples for each month, six samples were selected by random number generation in Excel, irrespectively whether the samples had previously tested human norovirus RNA positive or not. In total, fresh nucleic acids were obtained from 144 archived oyster proteinase K-treated digestive tissues.

### Nucleic Acid Extraction

For nucleic acid extraction, 500 μl of archived proteinase K-treated digestive tissue supernatants (stored at − 80 °C) was used as input for the NucliSENS Magnetic extraction kit (BioMérieux) according to manufacturer instructions. Nucleic acids were eluted in 100 μl buffer included in the kit. Two negative process control samples (NPC), PBS and proteinase K solution only, were run throughout the nucleic acid extraction stage per series of 12 samples. All selected samples met the criteria as described in ISO 15216–1:2017 for recovery (> 1%) and amplification controls (> 25%).

### Maxima H Minus Double-Stranded cDNA Synthesis

Double-stranded cDNA was generated using Maxima H Minus Double-Stranded cDNA Synthesis Kit (Thermo Fisher Scientific) according to the manufacturer’s instructions with minor modifications. In short, 250 pmol random hexamers were added to 11.5µL of nucleic acid extracts and incubated at 65 °C for 5 min. Subsequently, the first-strand master mix was added and the samples were incubated at 25 °C for 10 min followed by 30 min at 50 °C. The reaction was terminated by heating at 85 °C for 5 min and samples were stored on ice. After addition of the second strand synthesis master mix, the samples were incubated at 16 °C for 60 min. The reaction was terminated by the addition of 0.5 M EDTA and remaining RNA was removed by adding RNAse I and incubation for 5 min at room temperature. The double-stranded cDNA was then purified using 1.8 × volume AMPure XP beads (Beckman Coulter), after which the cDNA was eluted in 32µL Tris–HCl 10 mM pH 8.0 (Thermo Fischer Scientific).

### Library Construction

DNA libraries were generated using the KAPA HyperPlus Kit (Roche) according to manufacturer’s instructions with minor modifications. In short, DNA was enzymatically fragmented using Fragmentation Master Mix combined with 5 µL tenfold diluted Condition Solution at 37 °C for 10 min. After fragmentation, the samples were placed on ice. End repair and A-tailing Master Mix was added to each sample and incubated at 65 °C for 30 min. Ligation Master Mix and 5 µL 15 µM KAPA Unique Dual-Indexed Adapters (Roche) were added to each sample and incubated at 20 °C for 30 min, followed by a 1 × volume beads cleanup (AMPure XP, Beckman Coulter). Library Amplification Master Mix was added to each sample, followed by 10 amplification cycles in a thermocycler according to manufacturer instructions. After amplification, another 1 × volume bead-cleanup (AMPure XP, Beckman Coulter) was conducted and the DNA libraries were eluted in 50 µL Tris–HCl 10 mM pH 8.0 (Thermo Fischer Scientific). DNA was quantified using a Qubit HS dsDNA assay (Thermo Fischer Scientific). Subsequently, each series of samples (12 oyster sample libraries and 2 NPCs) were pooled, consisting of 167 ng per oyster sample plus 16.7 ng per NPC, roughly 2 µg in total for nucleic acid capture. For 144 oyster samples and 24 NPC’s, twelve hybridization captures were performed.

### Nucleic Acid Capture

The DNA library hybridization was performed using the KAPA HyperCap Workflow v3.0 (Roche) according to manufacturer’s instructions. COT Human DNA (1 mg/mL) was added to the pooled libraries, the samples were subsequently purified using 2 × volume AMPure XP beads with a single wash with 80% ethanol. After ethanol removal, Universal Enhancing Oligo’s were added, followed by the addition of Hybridization master mix, following the ≥ 40 Mbp capture target size workflow. After homogenization, samples were placed on a magnet and supernatant was transferred to the SeqCap EZ probe pool (VirCapSeq-VERT design) and incubated in a thermocycler at 95° for 5 min followed by 20 h incubation at 47 °C. After incubation, hybridized DNA was captured with beads and washed according to the manufacturer protocol (KAPA HyperCap Workflow v3.0; ≥ 40 Mbp capture target size) with minor adjustments. The capture beads were prepared and buffers were pre-warmed at 47 °C. The DNA was bound to the capture beads by a 15 min incubation at 47 °C. After capture, the beads plus probe-bound DNA were washed with stringent buffer at 47 °C, sequentially washed with wash buffer I, II and III. Enriched DNA was eluted in 20 µL PCR grade water. The post-capture PCR master mix was prepared followed by 13 amplification cycles using the manufacturer’s thermocycling protocol. After amplification a 1.4 × purification (AMPure XP, Beckman Coulter) was performed, followed by 0.65–0.85 × volume double-sided size selection. Samples were eluted in 35 µL Tris–HCl 10 mM pH 8.0 (Thermo Fischer Scientific). The size distributions of DNA libraries were determined using the Agilent High Sensitivity D5000 Screentape on a Tapestation 4150 and, if required, size selected to a fragment size range of 250–500 bp. The amplified and virus enriched sequencing libraries were pooled in an equimolar manner prior to paired-end 150 bp sequencing on Illumina NovaSeq. Market-ready oysters (Supplementary Figure S3) were sequenced on a single Illumina flow cell together with the respective NPCs (Supplementary Figure S4). Control experiments with spiked-in viruses were sequenced together with their respective NPCs, and separate from the unpurified bivalve molluscan shellfish batches and their NPCs.

### Viral Metagenomics Pipeline

A pipeline was designed to detect viral species in metagenomic, short read sequencing data. Paired end sequences were preprocessed using fastp version 0.23.2 (Chen et al., 2018), which involved tail end trimming (–trim_tail1, –trim_tail2), adapter trimming (–detect_adapter_for_pe) and dereplication (–dedup). Quality filtering and length filtering were performed at default settings. The processed reads were classified based on taxonomy using Kraken2 version 2.1.2 (Wood et al., 2019) in combination with a custom database containing the archaeal, bacterial, human, plasmid, protozoa, viral and core UniVec reference libraries downloaded from the NCBI RefSeq database (O’Leary et al., 2016), accessed on 2022–02-15, in addition to references for oyster (C. gigas GCF_902806645.1) and mussel (M. edulis GCA_019925275.1). Further analyses focused only on human infectious viruses included in the Virus-Host DB, retrieved on 2022–02-11 (Mihara et al., 2016). As Kraken2 has the tendency to overclassify reads on species level (Allnutt et al., 2021), a confirmation was performed by further sequence alignment. For this, reads classified by Kraken2 as part of a particular virus family were aligned with Bowtie2 version 2.4.4 (Langmead & Salzberg, 2012) (paired-end mapping with -a ‘match-all’ setting, other settings were default) against all available genomes for that family, downloaded from RefSeq, accessed on 2022–04-21. All valid alignments were reported (-a). Coverage information was retrieved using the BBMap tool pileup. Count data is deposited as supplementary information S1-4. Visualization was performed using altair. For Table 5 and 6, average genome coverage (± SD) across positive samples was calculated, virus species with average genome coverage above 5% in NPC’s were omitted from the main Tables. Subtyping was performed by comparison of reads to norovirus and sapovirus reference strains (Chhabra et al., 2019; Oka et al., 2015) using Sourmash version 4.8.2 (Pierce et al., 2019) (scale: 1000, threshold_bp: 50,000). The genome sequences of the spike-in viruses were added to the metagenome database for analyses of the control experiments only.

### Control Experiments and Quantification

Three fecal samples, positive for norovirus and characterized as genotype GI.2, GI.3 or GII.4, were kindly provided by Harry Vennema, RIVM, the Netherlands. Feline calicivirus culture supernatant was kindly provided by Erwin Duizer, RIVM, the Netherlands. Hepatitis A virus (HM 175, clone 1) was obtained from ATCC VR 1541; hepatitis E virus genotype 3c was extracted and concentrated from pig feces as described earlier (Boxman et al., 2017).

Control experiments were carried out on pooled proteinase K-treated digestive tissue supernatants that previously had tested negative for norovirus GI and GII RNA with RT-qPCR assays (ISO 15216–1:2017). To determine the sensitivity of the metagenomic detection with hybridization capture for norovirus RNA specifically, 500µL portions of proteinase K-treated digestive tissue supernatants were spiked in duplicate with a tenfold dilution series of GI.2 and GII.4 positive fecal samples. Following nucleic acid extraction, viral copy numbers were determined at 667, 63, 5.1, 1.19 and 0.08 gc/µL for norovirus GI and 708, 78.3, 7.5, 0.82 and 0.42 gc/µL for norovirus GII using reverse transcription digital PCR (RT-dPCR) on a QX200 platform (BioRad) as described previously (Boxman et al., 2024).

Another control experiment was performed to investigate the efficiency of the library preparation and hybridization capture for each of the viruses in a mixture of orthologous strains without or in a mixture with a complex matrix background. For this, 500µL portions of pooled proteinase K-treated digestive tissues, previously tested negative for norovirus GI, norovirus GII, hepatitis A virus (ISO 15261–1:2017) and hepatitis E virus (Jothikumar et al., 2006) by RT-qPCR assays, were spiked in quadruplicate, aiming at concentrations of 100–200 genome copies per µl in the final nucleic acid extract (2.2). In parallel, some of the samples were spiked with an abundance of feline calicivirus, aiming at a concentration of 2000 feline calicivirus genome copies per µl nucleic acid extract. Each spike dilution was also directly extracted in the absence of matrix. The final copy numbers of nucleic acids were determined by RT-dPCR assays. Viral input was 122 gc for human norovirus GI.2, GI.3 (144 gc), GII.4 (176 gc), hepatitis A virus (174 gc), hepatitis E virus 3c (198 gc) and feline calicivirus (2602 gc). A non-spiked proteinase K-treated digestive tissue was extracted and analyzed as negative process control. All samples underwent hybridization capture prior to sequencing analysis (Supplementary Figure S1).

A last control experiment was performed to investigate whether the assay and bioinformatic pipeline were able to identify genomic sequences of wild-type viruses from bivalve molluscs. For this, we selected 24 archived proteinase K-treated digestive tissues of undepurated oyster (*n* = 13) and mussel (*n* = 11) batches harvested at B-classified growing areas. The samples were prepared for hybridization capture prior to sequencing, as described above.

### Confirmation of Metagenome Targets Using (RT-)dPCR

Virus genome copies were quantified using (RT)-dPCR. The norovirus assay was performed as previously described (Boxman et al., 2024). Primer and probe sets for sapovirus were adapted from those published by Oka and colleagues (Oka et al., 2006), with a forward primer for genotypes (gt) 1, 2 and 4 and another one for gt 5. The primer probe sets for Aichi virus A and Aichi virus B strains (Kitajima et al., 2013) and for adeno-dependoparvovirus 2 (AAV2) (Aurnhammer et al., 2012) were collected from literature. Thermocycling of all assays was optimized for maximum genome copy recovery and fluorescence on a BioRad QX200 platform by testing a range of parameters for reverse transcription temperature, annealing temperature, primer and probe concentrations, as described earlier for the norovirus assays (Boxman et al., 2024) (Tables 1, 2, and 3).

**Table 1:** Oligonucleotides for (RT)-dPCR assays used in this study

Virus species	Primer/probe	Concentration	Sequence*
Sapovirus (adjusted from (Oka et al., 2006) to complement contemporary genotypes)	SaV124F-Adj	1500 nM	GAYCWNGCYCTCGCCACCT
	SaV1245R	1500 nM	CCCTCCATYTCAAACACTA
	SaV1245TP-Adj	750 nM	FAM-CCNCCWATRWACCA-MGBNFQ
	SaV5F-Adj	1500 nM	TWYGARCARGCBRYBGCRTGYTAY
Aichi virus A & B (Oka et al., 2006)	AiV-AB-F	900 nM	GTCTCCACHGACACYAAYTGGAC
	AiV-AB-R	900 nM	GTTGTACATRGCAGCCCAGG
	AiV-AB-TP	250 nM	FAM-TTYTCCTTYGTGCGTGC-MGBNFQ
Adeno-dependoparvovirus 2 (Aurnhammer et al., 2012)	AAV2-F-ITR	900 nM	GGAACCCCTAGTGATGGAGTT
	AAV2-R-ITR	900 nM	CGGCCTCAGTGAGCGA
	AAV2-P-ITR	250 nM	FAM-CACTCCCTC/ZEN/TCTGCGCGCTCG-IBFQ

^*^Degenerate bases follow IUPAC nucleotide codes. FAM, ZEN, MGBNFQ and IBFQ are fluorescence and quencher moieties

**Table 2:** dPCR reaction mixes

Sapovirus G124		Sapovirus G5	
Reagents	Volume (µL)	Reagents	Volume (µL)
1-step RT-dPCR mix (BioRad)	5	1-step RT-dPCR mix (BioRad)	5
Dithiothreitol (300 nM BioRad)	1	Dithiothreitol (300 nM. BioRad)	1
SaV124F-Adj (50 µM)(1500 nM)	0.6	SaV5F-Adj (50 µM)(1500 nM)	0.6
SaV1245R (50 µM)(1500 nM)	0.6	SaV1245R (50 µM)(1500 nM)	0.6
SaV1245TP-Eclips (5 µM)(750 nM)	3	SaV1245TP-Eclips (5 µM)(750 nM)	3
Nuclease free water	2.8	Nuclease free water	2.8
RT enzyme (BioRad)	2	RT enzyme (BioRad)	2
RNA	5	RNA	5

Aichi virus A & B		AAV2	
Reagents	Volume (µL)	Reagents	Volume (µL)
1-step RT-dPCR mix (BioRad)	5	ddPCR mix 2x (BioRad)	10
Dithiothreitol (300 nM. BioRad)	1	AAV2-ITR-F (10 µM)(900 nM)	1.8
AiV-AB-F (10 µM)(900 nM)	1.8	AAV2-ITR-R (10 µM)(900 nM)	1.8
AiV-AB-R (10 µM)(900 nM)	1.8	AAV2-ITR-P (5 µM)(250 nM)	1
AiV-AB-TP (5 µM)(250 nM)	1	Nuclease free water	0.4
Nuclease free Water	2.4	DNA	5
RT enzyme (BioRad)	2		
RNA	5

**Table 3:** RT-dPCR thermocycling

Step	Time	Target/Temp °C		
		Sapovirus G124 Sapovirus G5	Aichi virus A & B	Adeno-dependoparvovirus A
1	60 min (ramp rate 3 °C/s)	46	48	–
2	10 min (ramp rate 3 °C/s)	95	95	95
3	30 s (ramp rate 3 °C/s)	95	95	95
4	1 min (ramp rate 3 °C/s)	50	51	53
5	Go to step 3; 39x			
6	10 min (ramp rate 3 °C/s)	98	98	98
7	30 min (ramp rate 3 °C/s)	4	4	–
8	For ever (ramp rate 3 °C/s)	12	12	12

## Results

### Nucleic Acid Probe Hybridization Capture

For non-targeted identification of viral hazards to humans present in oyster gastrointestinal tissues, we aimed to characterize all vertebrate virus genomes present in these samples using massively parallel genome sequencing. For this, hybridization capture sequencing was performed targeting the vertebrate viral fraction (Briese et al., 2015). After library construction and probe hybridization capture, viral enrichment as determined by RT-qPCR was 53-fold for human norovirus GI as determined in duplicate by RT-qPCR in pre- en post-capture samples (ΔCq 5.73).

### Performance of Hybridization Capture Metagenomics for the Detection of Norovirus

**Fig. 1:**
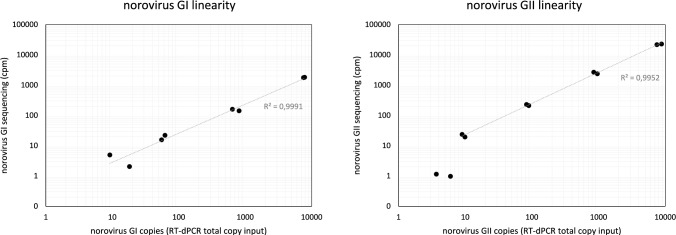
Linearity and pearson correlation between human norovirus genome copies and metagenome sequencing results. Results are shown for norovirus GI.2 (left) and norovirus GII.4 (right), with RT-dPCR measurements on the X axis and sequencing reads as ‘counts per million’ (cpm) on the Y axis

To determine the performance of the assay for specific foodborne viruses relevant to oysters in *hybridization captured metagenomic analysis*, digested oyster gastrointestinal tissue was spiked with norovirus genogroup GI.2 and GII.4 at varying concentrations. Nucleic acids were extracted and analyzed for viral genome copy (gc) numbers (determined by RT-dPCR) and compared with human norovirus specific reads after library construction, hybridization capture and sequencing. Sequencing read counts were normalized to ‘counts per million’ (cpm) in order to compensate for differences in sequencing depth between samples. Human norovirus GI and GII sequencing results were linear over 3Log_10_ dilutions of virus that was added to digested oyster gastrointestinal tissue (Fig. 1), with a slope of 1.03 (norovirus GII) and 0.96 (norovirus GI). The genome copies and cpm measurements correlated well (*R*^2^ > 0.995), suggesting the possibility of relative quantitation for both targets tested.

The lowest detection event in this tenfold dilution series was 14 gc for norovirus GI.2 and 5 gc for norovirus GII.4. At these levels, respectively 20% and 8% of the norovirus genome was covered. Entire genomes (> 97.5% compared to reference) were covered at on average 725 total gc input for norovirus GI.2 and 86 gc for norovirus GII.4 using the database including strain-specific genome sequences. Without the manual addition of reference genomes, the maximum coverage achieved by RefSeq was 31.8% for GI.2 and 59.6% GII.4 at the highest spike-in levels only.

### Hybridization Capture Metagenomics on Mixtures of Viruses in the Presence of Oyster Tissue

Next, we determined the performance of the capture-based metagenomic assay in samples containing mixtures of viruses with high sequence similarities, with identities of 60–20% between norovirus GI.2, GI.3, GII.4 and feline calicivirus relative to each other, in the presence and absence of proteinase K-treated oyster digestive tissue. In addition, samples were spiked with a high copy number of feline calicivirus or left un-spiked for feline calicivirus. Results of metagenomic sequencing are summarized in Table 4 and shown in Supplementary Figure S1. All viruses were retrieved with high genome coverage (cov) (93–100%) from the virus mixture in absence of shellfish matrix. Also, after extraction of most of the viruses from the digested oyster gastrointestinal tissue, the virus recovery was high, even though the viruses were spiked in low copy numbers. The presence of high gc of feline calicivirus did not influence the results for norovirus GI.2, norovirus GI.3 and norovirus GII.4, despite its sequence homology with these viruses. When viral nucleic acids were co-extracted from proteinase K-treated tissue, however, cpm and genomic coverage (cov) statistic values of all spiked viruses were negatively affected as compared to values for viruses analyzed in the absence of tissue. This could be due to less efficient extraction or hybridization capture of viral nucleic acids in the presence of matrix. However, in most cases, near-complete genomes could still be assembled, despite the very low amount of genome copies added to these samples. Assay reproducibility for genome coverage was high, with a coefficient of variation of 0–2.5% for nucleic acid controls and 0.1–11.1% in the presence of oyster background matrix. The coefficient of variation was best in those cases where the genomic coverage approached 100%.

**Table 4:** Effect of digested oyster gastrointestinal tissue and feline calicivirus on metagenomic sequencing of a virus mixture

	Capture-based metagenomic analysis of a virus mixture (*n* = 4)					
	No matrix, No feline calicivirus		No matrix, With feline calicivirus		Digested oyster gastrointestinal tissue, With feline calicivirus	
Virus (gc)	cpm ± CV%	cov ± CV%	cpm ± CV%	cov ± CV%	cpm ± CV%	cov ± CV%
Feline calicivirus (2602)	2 ± 0	3.9 ± 0	15993 ± 5	99.9 ± 0.1	3451 ± 11	99.9 ± 0.1
Norovirus GI.2 (122)	1296 ± 17	96.2 ± 2.5	1490 ± 1	97.3 ± 1.1	33 ± 28	79.8 ± 11.1
Norovirus GI.3 (144)	2010 ± 10	98.7 ± 1.6	1787 ± 7	98.3 ± 1.2	46 ± 9	76 ± 6.4
Norovirus GII.4 (176)	14657 ± 4	99.9 ± 0.1	14481 ± 6	99.7 ± 0.3	457 ± 14	99.5 ± 0.2
Hepatitis A virus (174)	6530 ± 7	99.1 ± 0.3	7067 ± 6	98.4 ± 0.5	116 ± 6	92.8 ± 2.6
Hepatitis E virus gt3c (198)	19284 ± 5	100 ± 0	19266 ± 15	99.9 ± 0.1	485 ± 10	99.7 ± 0.3

*gc* genome copies determined by RT-dPCR, *cpm* sequence read counts expressed as per million reads, *cov* percentage genome coverage, *CV*% coefficient of variation

**Fig. 2:**
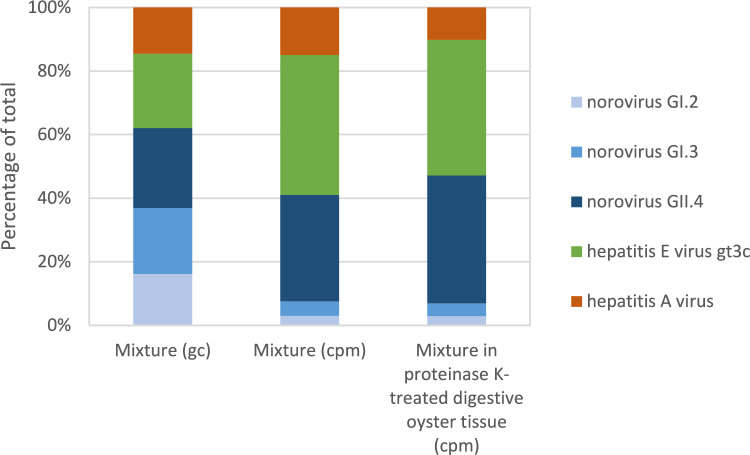
Ratio of viral genome copies or cpm after metagenomics. Mixture (gc) = gc of each virus as % of total viral gc as determined by RT-dPCR in the mixture without shellfish matrix; Mixture (cpm) = metagenome cpm for each virus as % of total cpm for virus spiked-in mixture; Mixture in proteinase K-treated digestive oyster tissue (cpm) = metagenome cpm for each virus as % of total retrieved from virus spiked oyster digested gastrointestinal tissue

Next, we inquired whether hybridization capture is able to capture various viral strains in the same ratio as they have been added (Fig. 2). Experimentally each virus strain in the mixture was added in roughly equal amounts of genome copies, but the relative sequence counts for each virus in the total mixture did not reflect this, nor in the presence nor absence of oyster digestive tissue background nucleic acids. Therefore, while measurements are reproducible and sensitive, the metagenomics method applied here is not suitable for relative quantitation *between* different viruses present in a sample. The relative abundance of metagenome counts was hardly affected by the presence of oyster gastrointestinal tissue nucleic acid extract (without and with oyster gastrointestinal background, norovirus GI.2 3% to 3%, GI.3 5% to 4%, GII.4 33% to 40%, hepatitis E virus 3c 44% to 43%, hepatitis A virus 15% to 10%).

The above described experiments were performed on oyster proteinase K-treated tissues, having well identified and quantified contamination levels. To show that the method is able to identify genomic sequences of viruses from bivalve molluscan shellfish without a priori knowledge, 24 bivalve molluscan shellfish harvested at B-classified growing areas prone to sewage contamination were analyzed. During analyses of the raw reads, only virus species that are infectious to humans according to the Virus-Host DB were retained. As an indicator of accurate taxonomic allocation, the obtained genomic coverage compared to the best matching reference genome is depicted (Table 5). Viruses with an average genome coverage of less than 5% were omitted from Table 5, but shown in Supplementary Figure S2. A broad range of viruses was identified, covering the spectrum of single and double-stranded RNA as well as DNA viruses. Adeno-associated dependoparvoviruses A and B and canonical gastroenteritis viruses, norovirus and sapovirus, were detected in the majority of samples (> 75%). These viruses are known agents that are infectious to humans. Other findings included astroviruses, rotavirus and parvoviruses. A higher coverage for astrovirus, bocaparvovirus and circoviruses was observed in six out of 24 samples, often of mussel origin, but not consistently in all mussel batches. The number of batches is, however, too small to generalize differences in observed virome contents between the species, harvested in different areas.

**Table 5:** Identification of viral nucleic acids in undepurated bivalve molluscs from B-areas using metagenome analyses (n = 24)

Virus species	Nucleic acid	Average genome coverage (% ± SD)	Samples positive (#/%)
Adeno-associated dependoparvovirus B	ssDNA	68.2 ± 30.1	22/91.7%
Adeno-associated dependoparvovirus A	ssDNA	37.7 ± 39.3	20/83.3%
Norovirus	ssRNA	27.1 ± 30.4	19/79.2%
Sapovirus	ssRNA	27.7 ± 25	18/75%
Rotavirus A	dsRNA	23.2 ± 9.6	12/50%
Human circovirus VS6600022	ssDNA	49.9 ± 29.1	11/45.8%
Primate bocaparvovirus 2	ssDNA	47.3 ± 33.6	9/37.5%
Astrovirus MLB1	ssRNA	38.3 ± 33.1	8/33.3%
Human astrovirus	ssRNA	47.3 ± 32.5	8/33.3%
Primate bocaparvovirus 1	ssDNA	44.5 ± 36.1	8/33.3%
Mamastrovirus 1	ssRNA	41.6 ± 30.8	7/29.2%
Human polyomavirus 5	dsDNA	13.4 ± 6.4	4/16.7%
Gyrovirus galga1	ssDNA	14.4 ± 6.4	3/12.5%
HMO Astrovirus A	ssRNA	12.6 ± 5	3/12.5%
Gyrovirus homsa3	ssDNA	12.0 ± 2.7	2/8.3%
Human picobirnavirus	dsRNA	13.9 ± 0	1/4.2%
Human fecal virus Jorvi2	ssDNA	8.4 ± 0	1/4.2%
Astrovirus MLB2	ssRNA	8.1 ± 0	1/4.2%

These results demonstrate that the developed wet-lab and informatic workflow is able to identify a wide range of viral nucleic acids from precursor food products, without a priori knowledge of viruses that are present.

### Identification of Viral Nucleic Acids in Market-Ready Oysters

Next, the presence of viral nucleic acids present in ready to consume raw oysters on the Dutch market was explored. For this, 144 randomly selected archival samples covering the November-February periods from the November 2015 to February 2021 were prepared for construction of sequencing libraries, hybridization capture and sequencing.

As before, sequencing reads were analyzed and filtered for specificity to human hosts as defined in the Virus-Host DB, then sorted for descending average genome coverage. The latter, as high genomic reconstitution compared to the best matching reference species provides confidence in accurate taxonomic classification. Samples with an average percentage of reference genome covered above 5% are shown in Table 6. The comprehensive list can be inspected in Supplementary Figure S3.

**Table 6:** Presence of viral nucleic acids in market-ready oysters, sampled in the November-February periods from November 2015- February 2021 (n = 144)

Virus species	Average genome coverage (% ± SD)	Samples positive (#/%)	Human disease	Genome
Human associated cyclovirus 4	36.6 ± 20.5	2/1.4%	Association gastroenteritis & central nervous system disease	ssDNA
Adeno-associated dependoparvovirus B	23.9 ± 21.7	48/33.3%	Not clearly established	ssDNA
Human circovirus VS6600022	23.2 ± 8.9	3/2.1%	Not clearly established	ssDNA
Human associated cyclovirus 6	18.5 ± 5.5	3/2.1%	Association gastroenteritis & central nervous system disease	ssDNA
Gemycircularvirus HV-GcV1	18.4 ± 12.8	21/14.6%	Not clearly established	ssDNA
Adeno-associated dependoparvovirus A	14.2 ± 19.2	61/42.4%	Association with hepatitis	ssDNA
Gemykibivirus humas1	14.0 ± 8.9	32/22.2%	Not clearly established	ssDNA
Gemyvongvirus humas1	12.5 ± 6.8	6/4.2%	Not clearly established	ssDNA
Human picobirnavirus	10.8 ± 2.2	2/1.4%	Association with gastroenteritis	dsRNA
Norovirus	9.9 ± 13.1	63/43.8%	Gastroenteritis	ssRNA
Sapovirus	9.9 ± 12.5	43/29.9%	Gastroenteritis	ssRNA
Gyrovirus homsa3	7.4 ± 0	1/0.7%	Not clearly established	ssDNA
Gammapapillomavirus 11	5.7 ± 3	3/2.1%	Warts, papilloma	dsDNA
Aichi virus B	5.7 ± 5.1	16/11.1%	Gastroenteritis	ssRNA
Aichi virus A	5.1 ± 4.7	8/5.6%	Gastroenteritis	ssRNA

The fifteen viral hazards identified vary from well-established risk to humans to unclear pathogenicity to humans and are shortly outlined in Box 1. All viruses are non-enveloped, suggesting increased environmental stability compared to enveloped viruses.

**Box 1:** Viral nucleic acids detected in market-ready bivalve molluscs and their association with human disease

Norovirus and sapovirus cause acute gastroenteritis and are known for their significant impact on public health globally due to their ability to cause widespread outbreaks of gastroenteritis, particularly in semi-closed environment such as cruise ships, hospitals and schools (Patel et al., 2009), but also through retail establishments (Duret et al., 2017) and through food such as bivalve molluscs (BIOHAZ, 2012). Other viruses that were detected in oysters include *Picornaviridae* Aichi virus A and Aichi virus B and human picobirnavirus. Aichi viruses have emerged in recent years as responsible for gastroenteritis outbreaks associated with different foods (Rivadulla & Romalde, 2020). Multiple studies have determined a seroprevalence in the human population well over 50% in adults, suggesting widespread exposure to the virus. Human picobirnaviruses have been detected in fecal samples from gastroenteritis patients, often co-infected with other enteric viruses and bacteria (Baányai et al., 2003; Rivadulla & Romalde, 2020), but also in fecal specimens from disease-free humans. The pathogenicity of picobirnaviruses is not yet clearly determined and it may act as an opportunistic enteric virus in immunocompromised and co-infected individuals. An unexpected finding was skin-contact transmitted *Papillomavirus* gammapapillomavirus 11. Although historically associated with conditions such as skin warts and papillomatous lesions, it is now known that gammapapillomavirus 11 can be present in the oral cavity (Bottalico et al., 2011) and has been associated with head and neck cancer (Agalliu et al., 2016). Viruses with unclear pathogenicity to humans include *Circoviridae* human-associated cyclovirus 4 (CyCV-4), human-associated cyclovirus 6 (CyCV-6) and human circovirus VS6600022; *Genomoviridae* gemycircularvirus HV-GcV1, gemykibivirus humas1 and gemyvongvirus humas1; *Anellovirus* gyrovirus homsa3 (GyV); and *Parvoviridae* adeno-associated dependoparvovirus A (AAV-A) and adeno-associated dependoparvovirus B (AAV-B). These viruses are either recently discovered or not well-characterized, with limited evidence regarding their pathogenicity in humans. For example, CyCV-4 and CyCV-6 have been detected in stool and cerebrospinal fluid and are associated with enteric and neurological diseases, but further study is needed to better understand their exact pathogenicity and infectivity (ECDC, 2013). Similarly, human circoviruses have been associated with idiopathic disease (Smits et al., 2014), although their seroprevalence in the healthy population is not yet known. Gemycircularviruses, gemikibivirus and gemyvongvirus belong to the *Genomoviridae* family of ~ 2.2 kb single stranded circular DNA viruses. Gemycircularvirus has been detected in human samples and in pathological context (Elbasir et al., 2023; Halary et al., 2016), although its contribution to disease is not yet clearly established. Gemikibivirus and gemyvongvirus are commonly detected in the blood of healthy donors, therefore these viruses are commonly referred to as commensals (Kandathil & Thomas, 2024). Few studies have suggested an association with disease which could warrant further investigation (Breitbart et al., 2017; Wang et al., 2019). Human gyroviruses have been detected in human blood (Maggi et al., 2012), skin (Sauvage et al., 2011) and feces (Sauvage et al., 2011), but no definitive relation with human disease has been established. Lastly, adeno-associated dependoparvoviruses have been utilized extensively in gene therapy and were considered non-pathogenic (Samulski & Muzyczka, 2014). However more recently, adeno-associated virus A2 presence in plasma and liver samples has become associated with an outbreak of acute pediatric hepatitis (Ho et al., 2023). Adeno-associated virus B is mostly associated with cattle. Although annotated as such in the Virus-Host DB, strong indications of AAV-B infectivity to human hosts could not be inferred from the contemporary body of literature.		

To check for potential sample cross-contamination during sample collection and preparation, negative process control (NPC) samples were incorporated in each hybridization capture. NPC results are shown in Supplementary Figure S4. It is worth noting that contamination in viral metagenomic assays can stem from a range of internal and external sources, including laboratory personnel, air contaminants, kit contaminants, laboratory equipment sequencing errors or sequencing index switching (Jurasz et al., 2021). In addition to detection in market-ready oysters, norovirus has been observed in one NPC (15 reads in NPC vs. an average of 44 (range 2–746) in samples) at 3.5% genome coverage and human polyomavirus 5 has been detected in seven NPCs (range 16–148 vs. 2–74 reads in samples) at 5.9% coverage. Caution is warranted for interpretation of pathogenic hazards that are identified in NPCs. Polyomavirus 5 was present in the sets of market-ready oysters and NPCs at a genome coverage above 5%, and therefore removed from Table 6. Retaining these identified sequences in the data set may lead to false positive results, whereas removing them may lead to false-negative results. At the hybridization capture stage, the sequencing libraries were pooled based on their DNA content, potentially resulting in an overrepresentation of NPC results.

### Verification of Targets

Next, a targeted method was used to confirm findings by metagenomic analysis. Targets were (again) selected based on their contribution to the global disease burden. (RT-)dPCR was applied to all samples against norovirus GI, norovirus GII, sapovirus 1–5, adeno-dependoparvovirus A and Aichi virus A and B species. Norovirus GI and norovirus GII genogroups are highly divergent and are therefore routinely detected using separate PCR assays: (RT-)dPCR copy number measurements for both genogroups were summed in order to relate them to metagenome cpm at the species level. Similarly, sapovirus 1–4 and sapovirus 5 were detected separately and copy numbers were summed. The primers and probe pair used for Aichi virus was specific for both Aichi virus A and B, therefore metagenome counts were summed for both species. After analyses, the percentage of samples positive for each virus species both after sequencing and in digital (RT-)dPCR, was calculated. The method correspondence between metagenomic analysis and (RT-)dPCR for norovirus was 63.5% (40/63 positive with both methods versus positive in sequencing). Sapovirus, adeno-dependoparvovirus A, and Aichi virus showed a correspondence of 79.1%, 63.9% and 57.1%, respectively (34/43, 39/61, 12/21). Some samples were positive in (RT-)dPCR only at very low concentration (norovirus 4.2%, sapovirus 9.5%, adeno-dependoparvovirus A 20.1%, Aichi virus 2.1%). It is important to note that the lowest detection event in the tenfold dilution series was 14 gc for norovirus GI.2 and 5 gc for norovirus GII.4 as total input in library preparation and using the in-house GI.2 and GII.4 viral sequences for mapping of the reads. The metagenomic detection sensitivity for wild-type viruses is dependent on the similarity with the reference strains used. Moreover, confirmation of the metagenome findings at low contamination levels is challenged by stochastic sampling in both assays. Despite these caveats, the relatively high correspondence between both methods under this condition demonstrates high sensitivity of both metagenome and (RT-)dPCR measurements.

### Virus Subtyping

To further confirm positive findings and better understand strain diversity retrieved in this set of market-ready oyster samples, viral subtyping was performed compared to strain reference sets. For norovirus GI and GII, both the capsid and polymerase regions were targeted. Sapoviruses were subtyped on the capsid region only. Results are depicted in Table 7. For Aichi virus and adeno-dependoparvovirus A, a suitable consensus set of reference genomes could not be identified from the literature.

**Table 7:** Subtyping of norovirus GI and GII (capsid and polymerase) and sapovirus (capsid)

Norovirus capsid type (# samples)	Norovirus polymerase type (# samples)	Sapovirus capsid type (# samples)
GII.4 (15)	GII.P16 (7)	GIII (21)
GII.17 (4)	GII.P31 (5)	GII.2 (8)
GII.2 (3)	GI.P4 (2), GII.P4 (2)	GI.2 (6)
GII.6 (2)	GII.P17 (4)	Not assigned (6)
GI.4 (1)	GII.P12 (3)	
GII.9 (1)	GII.P7 (2)	
GII.13 (1)	GII.P2 (1)	
Not assigned (36)	GII.P21 (1)	
	Not assigned (36)	

43% of samples with norovirus detected were subtyped at the capsid level. Of these, norovirus GII.4 was most common (55%), which corresponds to earlier reports describing the prevalence and epidemic potential for GII.4 strains (Carlson et al., 2024). Other norovirus capsid types detected were GII.17, GII.2, GII.6, GII.9 and GII.13. A single GI genotype was detected at the capsid level, GI.4. A range of norovirus polymerase types was detected: GII.P16, GII.P31, GII.P17, GI.P4, GII.P4, GII.P12, GII.P7, GII.P2 and GII.P21. The majority of sapovirus was typed as GIII (60%) in the capsid region. Other sapovirus capsid types detected were GII.2 and GI.2.

## Discussion

We have applied a hybridization capture-based viral metagenomics workflow on oysters to screen for known and unknown vertebrate viral RNA and DNA genomes. The assay showed high sensitivity and linear relationship between the spiked-in norovirus GI.2 and GII.4 RNA copy numbers and the obtained reads from proteinase K-treated oyster digestive tissue. Application of the method to undepurated bivalve molluscs, as well as to a large number of market-ready oyster batches, identified a range of viruses, including human norovirus, sapovirus, and Aichi virus. A follow-up study with targeted RT-dPCR assays showed that viral genomic copy numbers in market-ready oyster batches were low. Metagenomic analysis further identified a number of potential pathogenic hazards in market-ready bivalve molluscs, based on the genomic signatures that matched virus species with evidence of infectivity in humans. We identified an abundance of genomic fragments from picobirnavirus, multiple *Genomoviridae*, gyrovirus cyclovirus and adeno-dependoparvoviruses. These viruses represent hazards with a potentially under-acknowledged disease burden in humans, though from the current data it is not possible to extrapolate whether the viral genomic fragments are derived from viruses that can lead to foodborne infections in humans. Therefore, this study warrants further investigations to determine the importance and infectivity of these viruses in food, such as bivalve molluscs.

Earlier metagenome studies in oysters were performed with a focus on norovirus contamination in either a small set of six oysters (Tan et al., 2021), in laboratory bioaccumulated oysters not meant for human consumption (Strubbia et al., 2020), or using laboratory-prepared samples of known norovirus composition (Ollivier et al., 2022). The same VirCapSeq-VERT assay as in the present study was used previously (Bonny et al., 2021) to investigate the vertebrate virome diversity in fourteen fresh water Camaroonian clam samples, which are usually consumed after heat treatment. The VirCapSeq-VERT assay was also used to create a ‘Dataset of the total Oyster Virome’ from 35 oyster samples in the context of marine virus exploration, rather than placing focus on the human public health aspects (Jiang et al., 2023).

For a better understanding of the performance and limitations of the hybridization capture-based metagenomics workflow, first a titration experiment was performed on proteinase K-digested oyster tissue with spiked-in human norovirus GI.2 and GII.4. The metagenome counts had a linear relationship with (RT-)dPCR measurements for both norovirus targets, however, the metagenomics method applied was more sensitive for norovirus GII.4 even when both strains were added to the reference database. We hypothesize that this difference might relate to a smaller size of the GI probe pool compared to GII, or that the spiked-in virus has a reduced sequence identity to the GI probe pool. We are not able to exclude either possibility as the probeset in the VirCapSeq-VERT panel is not fully disclosed. We note that the detection sensitivity for wildtype noroviruses could differ depending on the sequence similarity with the best matching reference strain available in the selected database.

As bivalve molluscs most likely contain mixtures of viruses, we subsequently applied the assay to a pool of viruses of known concentrations in the presence or absence of oyster matrix. We conclude that the presence of more abundant feline calicivirus RNA in the mixtures, which is used as process control virus during extraction of bivalve molluscs, did not hinder the metagenomic detection of multiple lower abundant viruses in the sample. Compared to targeted RT-dPCR measured input, norovirus GI.2 and GI.3 were underrepresented in the metagenomics data of the mixture experiment. Again, this observation may be related to (a combination of) the hybridization probe pool complexity that is not equally sized for each virus strain and/or the sequence identity of the probes with the virus target. Although results did not allow for inter-viral quantitative comparison, sequence counts were retrieved for all viruses spiked at low copy numbers (98–152 copies per reaction for norovirus GI.2, GI.3, GII.4, hepatitis E virus, hepatitis A virus). A similar approach was done in a study with a focus on mixture of different norovirus genogroups and types and two laboratories (Ollivier et al., 2022) showing detection of the full diversity of the spiked strains at four to eight thousand copies per µL, though a subset of the virus strains was retrieved using hybridization capture metagenomics at lower copy number inputs.

Finally, we confirmed that the chosen workflow is able to detect and identify the wide range of vertebrate viral genomic nucleic acids in bivalve molluscs harvested from growing areas prone to sewage contamination. Regulation (EC) No 853/2004 requires that bivalve molluscs harvested from such Class B locations either undergo microbial inactivation, relaying or depuration before commercial sale for human consumption. In earlier research, members of the *Astroviridae, Picornaviridae, Picobirnaviridae, Caliciviridae,* and *Reoviridae* families have been detected in sewage samples and sewage experimentally bioaccumulated oysters (Strubbia et al., 2019). In the present virome study, members of these families (except for *Picornaviridae*) were detected in the undepurated B-class samples as well.

With this affirmation, the method was ready for application on our selected sample set. To our knowledge, this is the largest study to apply metagenome analyses for the presence of vertebrate viruses in market-ready oysters. The samples were collected from the Dutch market in November up to and including February over a window of 6 years. The metagenomic analyses revealed many viruses, besides the more commonly described norovirus, also the presence of rotavirus, multiple species of astrovirus and bocaparvovirus, as well as sapovirus, adeno-associated dependoparviruses, picobirnavirus, gyrovirus and circovirus. Ineffectiveness of depuration for removal of viruses has already been shown previously for norovirus and hepatitis A virus (Battistini et al., 2021; Heller et al., 1986; Love et al., 2010; Martinez-Albores et al., 2020; McLeod et al., 2017; Polo et al., 2014; Rupnik et al., 2021). Depuration properties for other virus species are unknown, warranting a study design with matched samples taken before and after depuration, making this a topic for future research. We acknowledge that an unknown portion of the viral nucleic acids may have been degraded during the proteinase K treatment of the oyster tissues, and no longer be available for metagenomic detection.

Presence of norovirus, sapovirus (Varela et al., 2016), and Aichi virus RNA (Rivadulla & Romalde, 2020), well known agents of gastroenteritis transmitted through food, have been reported earlier in bivalve molluscs. The metagenome results, with read counts near the end of the titration curve for norovirus GI.2 and GII.4, suggested that the norovirus concentration in this set of 144 oysters was low, in line with earlier observations (Dirks et al., 2021). Still genomic coverage was above ten percent in seventeen samples, ranging up to 70%. Moreover, genotyping was possible for 43% of these samples. Sapovirus and Aichi virus copy numbers in the market-ready oysters were comparably low.

The presence of picobirnavirus dsRNA has only recently been described in a study of fresh river water Cameroonian clams, with strains infecting humans, bats, and gorilla (Bonny et al., 2021). The present study is, however, the first report describing detection of human-specific adeno-associated dependoparvovirus species DNA in market-ready oysters. Adeno-associated dependoparvoviruses (AAV) are small viruses that depend on the presence of a helper virus, such as adenovirus, for their replication in the host cell. AAV2 viruses gained recognition as an emerging human pathogen (Ho et al., 2023) after demonstration of AAV2 infection in children with severe non-A–E hepatitis in April 2022 in Scotland. Adenovirus DNA itself, however, was not detected within the tested oyster samples. Nucleic acids from other viruses likely to represent viruses with a potential human disease burden are members of the *Circoviridae*; circovirus (Smits et al., 2014) and cyclovirus (ECDC, 2013). Circoviruses in oysters have been reported (Jiang et al., 2023), but not in a human clinical context. Nucleic acids of other viruses were gemycircularvirus (Elbasir et al., 2023; Halary et al., 2016), gemykibivirus and gemyvongvirus (Breitbart et al., 2017; Wang et al., 2019). As this study focused on the hazard identification of viral nucleic acids in market-ready oysters using molecular tools only, risk assessment for our findings remains a topic for future research.

For allocation of massively parallel sequencing reads, an in-house computational pipeline was applied. To achieve the highest method sensitivity possible (Schönegger et al., 2023), we applied a two-stage read mapping strategy at the species taxonomic level, rather than an assembly-based procedure. The Virus-Host DB, that was used to select for virus species that are infectious to humans, performed fit for purpose, as no clearly non-human virus species were retrieved at meaningful sequencing coverage. In addition to such a well curated target species databases, good reference genome datasets are paramount to perform future metagenomic assays. In this study, viral reads were matched to best matching RefSeq sequences. For norovirus spiked at high genome copy numbers, mapping did not exceed 60% of genome coverage, whereas mapping to the specific norovirus GI.2 and GII.4 sequences did. This demonstrated that for norovirus the RefSeq collection was not optimal, which may be true for other viruses as well. Improvement of reference databases is of ultimate importance to harness the full potential of viral metagenomics, as well as databases for strain-level subtyping, as we could pinpoint reference genome sets for norovirus and sapovirus only.

One of the analytical complexities in metagenome studies is the data cutoff. Table 5 and Table 6 include only virus species with a minimum average genome coverage of 5% across *all* positive samples. The criterium was based on the results of the negative extraction controls (NPC) that were run in parallel during the whole process and were 2.7% ± 2.6 average genome coverage across all false positive findings (Supplementary Figure S4).

Viruses such as norovirus, sapovirus, hepatitis A virus and Aichi virus are known for their prolonged environmental stability. Detection of nucleic acids in virome studies, as for targeted molecular detection, does not proof that detected nucleic acids are derived from infectious particles with a human health risk after consumption of raw oysters. This emphasizes a clear need for efficient and cost-effective infectivity screens of these viruses. Despite recent advances in technology, providing proof-of-infectivity on foodborne disease remains challenging, and is not yet amenable to high throughput. Ettayebi and colleagues (Ettayebi et al., 2016) have shown replication of some norovirus genotypes in human enteroids, though the assay has not yet been proven effective for all strains. Others have used the system for different viruses (Kolawole et al., 2019). Amplification of hepatitis E virus using primary cells and a cell line has been demonstrated previously (Honing et al., 2023; Johne et al., 2013; Stunnenberg et al., 2023). However, for many novel targets detected in this study, standardized infectivity assays do not yet exist. Other challenges are to maintain the infectivity of the viruses during extraction from the matrix, the concentration of viruses into the required viral input, and cost-effectiveness.

Until in vitro viral infectivity assays become widely available, the information obtained from viral metagenomic sequencing can still provide guidance if correlated to clinical data. Matching of food related pathogen genome data with that of a genomes detected in (hospitalized) patients provides a further indication of infectivity, especially when an epidemiological link can be established. To bridge the proof-of-infectivity gap and until infectivity assays become available, sharing of pathogen genomic sequences between hospitals and food safety laboratories is essential. Food is not always available for analyses in foodborne outbreaks, because it has been consumed or discarded.

Bivalve molluscan shellfish were chosen for this study as they are known to bioaccumulate viruses, and are often consumed raw. A limitation of the present study is that only in November up to and including February were sampled, as this is the period in which most oysters are consumed locally, and the months coincide with the viral gastroenteritis season. The presence of viral nucleic acids in market-ready oysters on the Dutch market in the other months (March-October) remains a knowledge gap, as different viruses circulate with distinct seasonal patterns (e.g., certain enteroviruses during summer months).

In summary, we have investigated a large set of market-ready oysters using viral metagenomic sequencing. In addition to known viruses, we detected a range of viral nucleic acids that belong to virus species infectious to humans, collectively harboring a potential disease burden. We show the potential of metagenomics for food product hazard identification envision a role for metagenome analysis in pathogen source tracking and for epidemiological investigation of foodborne outbreaks. Over the last few years, multiple developments have expanded the capacity for viral enrichment and sequencing in laboratories worldwide. Increased pathogen data sharing and improved reference databases are needed to best utilize the metagenome data that is being generated. By integrating metagenome analysis into the monitoring process, researchers and regulatory bodies can gain valuable insights into the viral and communities present in foods and in the environment. By leveraging metagenomics for the detection of potential pathogenic hazards in food products, an opportunity arises for well-tailored risk-based monitoring programs and targeted interventions to maintain the sanitary quality of foods and production areas and to safeguard public health.

## Supplementary Information

Below is the link to the electronic supplementary material.

Supplementary file1 (HTML 26 KB)

Supplementary file2 (HTML 133 KB)

Supplementary file3 (HTML 320 KB)

Supplementary file4 (HTML 35 KB)

Supplementary file5 (DOCX 15 KB)

## Data Availability

All unfiltered virome results are provided within the supplementary information files (S1-S4) with descriptions (S5).
