# Supplementary material for: A Metagenomic Survey of Virological Hazards in Market-Ready Oysters
Source: Food Environ Virol. 2025 Jan 10;17(1):16. doi: 10.1007/s12560-024-09630-2 (PMC11723887; doi:10.1007/s12560-024-09630-2)
Supplement: Supplementary file 5 — Supplementary file5 (DOCX 15 KB) [file 12560_2024_9630_MOESM5_ESM.docx]

# Supplementary data description

Supplementary Figure S1: Complete data, virome analysis of mixed virus spike-in experiment. Abbreviations: A/B/C/D = replicates. Non-spiked-OysProK = oyster proteinase K-treated tissue, only FCV virus added as extraction control. Ratio = GI.2, GI.3, GII.4, HAV and HEV3c virus mixture. RatioFCV = virus mixture with added FCV. Ratio-OysProK = virus mixture added to oyster proteinase K-treated tissue. Covered_percentage = the fraction of the genome covered when mapping reads to the specific reference genome. Average_fold = the average read depth for the covered fraction. CPM = read counts per million sequenced reads. Reads = virus species read counts.

Supplementary Figure S2: Complete data, virome analysis of 24 class-B bivalve molluscs. Covered_percentage = the fraction of the genome covered when mapping reads to the best matching species reference genome. Average_fold = the average read depth for the covered fraction. CPM = counts per million sequenced reads. Reads = total read counts. Oyster and mussel samples are labeled ‘O’ or ‘M’, respectively.

Supplementary Figure S3: Complete data, virome analysis of 144 market-ready oysters. Covered_percentage = the fraction of the genome covered when mapping reads to the best matching species reference genome. Average_fold = the average read depth for the covered fraction. CPM = counts per million sequenced reads. Reads = total read counts.

Supplementary Figure S4: Complete data, virome analysis of negative process control samples.

Covered_percentage = the fraction of the genome covered when mapping reads to the best matching species reference genome. Average_fold = the average read depth for the covered fraction. CPM = read counts per million sequenced reads. Reads = total read counts.
